# Genetics, genomics, and diet interactions in obesity in the Latin American environment

**DOI:** 10.3389/fnut.2022.1063286

**Published:** 2022-12-01

**Authors:** Patricia Guevara-Ramírez, Santiago Cadena-Ullauri, Viviana A. Ruiz-Pozo, Rafael Tamayo-Trujillo, Elius Paz-Cruz, Daniel Simancas-Racines, Ana Karina Zambrano

**Affiliations:** ^1^Centro de Investigación Genética y Genómica, Facultad de Ciencias de la Salud Eugenio Espejo, Universidad UTE, Quito, Ecuador; ^2^Centro de Investigación en Salud Pública y Epidemiología Clínica (CISPEC), Facultad de Ciencias de la Salud Eugenio Espejo, Universidad UTE, Quito, Ecuador

**Keywords:** obesity, nutrigenetics, nutrigenomic, Latin America, SNPs

## Abstract

Obesity is a chronic disease characterized by abnormal or excessive fat accumulation that could impact an individual’s health; moreover, the World Health Organization (WHO) has declared obesity a global epidemic since 1997. In Latin America, in 2016, reports indicated that 24.2% of the adult population was obese. The environmental factor or specific behaviors like dietary intake or physical activity have a vital role in the development of a condition like obesity, but the interaction of genes could contribute to that predisposition. Hence, it is vital to understand the relationship between genes and disease. Indeed, genetics in nutrition studies the genetic variations and their effect on dietary response; while genomics in nutrition studies the role of nutrients in gene expression. The present review represents a compendium of the dietary behaviors in the Latin American environment and the interactions of genes with their single nucleotide polymorphisms (SNPs) associated with obesity, including the risk allele frequencies in the Latin American population. Additionally, a bibliographical selection of several studies has been included; these studies examined the impact that dietary patterns in Latin American environments have on the expression of numerous genes involved in obesity-associated metabolic pathways.

## Introduction

Obesity is a chronic disease characterized by abnormal or excessive fat accumulation that could impact an individual’s health. The World Health Organization (WHO) defines adult obesity as when the body mass index (BMI) is equal to or greater than 30 kg/m^2^. Moreover, the WHO declared obesity a global epidemic in 1997 (1).

Additionally, obesity is considered one of the principal causes of morbidity and mortality in most countries. Worldwide, the prevalence of obesity has increased during the last decades; about 13% of the adult population was obese in 2016 (1). If this increasing trend continues, it is expected that the majority of the adult population will be obese by 2030 (2).

Obesity has become a health concern in Latin America, reporting a 24.2% of the region’s adult population was obese in 2016 (3). The highest prevalence was found in Argentina (28.3%), and the lowest was in Peru (19.7%). The data showed that the southern part of South America is more obese than the north (2, 4–6). Besides, the studies suggested that by 2030 up to 81.9% of the Latin American adult population may be obese or overweight (7).

The adverse impacts of obesity increase the risk for many diseases and health conditions, such as type 2 diabetes, high cholesterol, coronary heart disease, breathing problems, hypertension, stroke, and depression, among others (8–10). These conditions could cause disability and even premature death. Due to the lack of effective interventions for obesity prevention and management, the increase in obesity is not surprising, even with the socioeconomic disparities, because the incidence has gradually shifted to the lower-income population (11).

Genes could define susceptibility to a condition or disease, and the environmental factor or specific behaviors like dietary intake or physical activity could determine the development of that condition or illness (12). Studies of dietary patterns have been performed in North Americans and Europeans, but there is still a lack of evidence of food intake in Latin America (13). Latin American countries have been experiencing a change in eating habits, which has meant that the prevalence of undernutrition is declining, while the prevalence of overweight and obesity is increasing (7).

Moreover, a study published by Sepulveda said that the caloric intake varies from 1,880 to 2,170 calories/day, with an average animal protein consumption of 496 calories (14). In contrast, nowadays, the diet has changed; for example, a study by Kovalskys et al. reported 1,959 kcal/d divided into 54% carbohydrate, 30% fat, and 16% protein. Also, they described that the dietary intake of Latin Americans is based on grains, pasta, bread, meat, eggs, oils, fats, non-alcoholic beverages, and ready-to-drink beverages. Additionally, they reported that 25% of the energy intake comes from food rich in sugar and fat; meanwhile, 13% comes from fiber and micronutrients (13).

**FIGURE 1:**
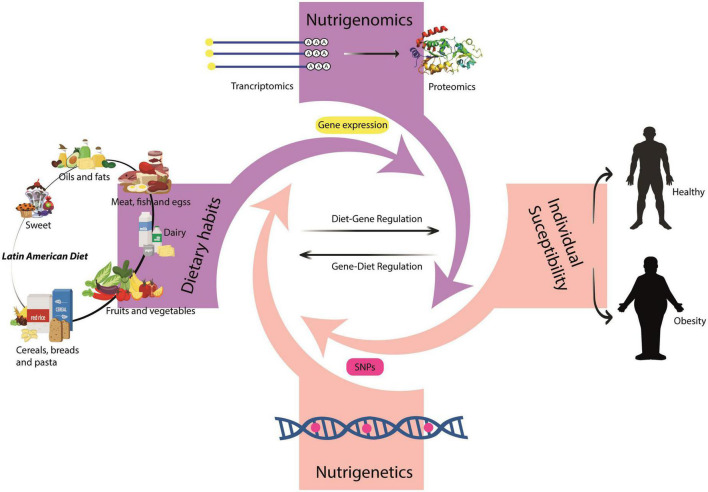
Nutrigenetics, nutrigenomics, dietary habits, and individual susceptibility. Nutrigenetics studies genetic variations (SNPs) and their effect on dietary response. Nutrigenomics studies the role that the nutrients have on gene expression with a focus on the Latin American environment. Collectively, these three factors could increase or decrease an individual’s susceptibility to develop obesity in Latin America.

Nutrigenetics and nutrigenomics provide data related to the mechanism of nutrients and genes interactions, approaches for precision nutrition, and their relation to disease risk (Figure 1). Indeed, nutrigenetics studies genetic variations and their effect on dietary response (12). The genetic variations are DNA sequence differences between individuals or populations, including single nucleotide polymorphisms (SNPs) and copy number variations (CNV). Those genetic variants could modify the effects of dietary intake, affect food metabolism, and influence food preferences. Meanwhile, nutrigenomics studies the role of nutrients in gene expression (15). As a matter of fact, diet intake could directly affect gene expression and genome stability, leading to diseases or adverse phenotypes in any life stage (16).

The Latin American population constitutes a mixed population of Native American, European, and African descendants (17). Ethnicity could influence the genetic predisposition to obesity and dietary intake could affect gene expression. The present study constitutes a complete review of the dietary behavior in the Latin American environment related to obesity. It is divided into two main sections, the first one “the genetics in obesity in the Latin American population,” which describes the genetic variants associated with food intake in Latin America; and the second section “Genomics and diet interaction in obesity in the Latin American environment” that presents how nutrition could impact the gene expression influencing the incidence of obesity in the population.

## Genetics in obesity in the Latin American population

As described in the introduction, genetics in nutrition is the science that studies the interactions between genetic variations and how the body processes nutrients, associating the variation with human health and disease, including obesity (18, 19). Moreover, obesity is increasing worldwide, and Latin America is no exception (4). Hence, it is vital to understand the interaction between genetic variation and obesity in the Latin American population due to the differences in ethnicity with other regions.

**FIGURE 2:**
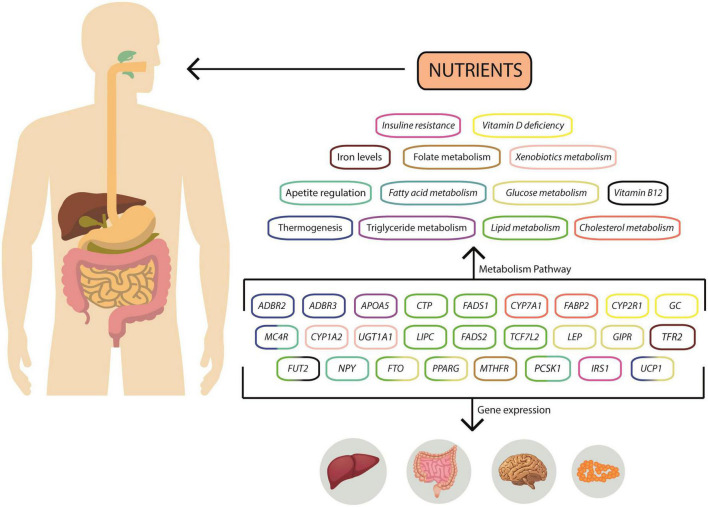
Overview of gene interactions and gene expression in nutritional metabolism pathways. Diet can influence gene expression in several ways, and nutrients can be metabolized through various pathways, altering the concentration of substrates or intermediates that influence gene expression. Changes in gene expression can impact the liver, hypothalamus, and adipose tissue. These nutrient-gene interactions can be detrimental, increasing the risk of obesity.

The present section represents a compendium of genes and their SNPs associated with obesity (Figure 2). Moreover, the Latin American population’s reference and alternative allele frequencies were retrieved from the ALFA: Allele Frequency Aggregator and The Population Architecture using Genomics and Epidemiology (PAGE) Study. The ALFA: Allele Frequency Aggregator is a database that contains over two million subjects, from different ancestral backgrounds, including African, Asian, European, Latin American, and others (20). Similarly, The PAGE Study is another database that contains allele frequencies; however, this project is focused on describing the genetic components of underrepresented minorities (21). The terms included in the review included Latin American, South American, and Native American. Additionally, to describe the function of each gene, its association with environment, and obesity, the GO (Gene Ontology) Biological processes were included.

Table 1 describes the allele frequencies, in Latin America, of SNPs associated with obesity. Even though most of the studies correlating SNPs and obesity predisposition have been performed on Asian and European populations, identifying the frequency of these risk alleles, in Latin America, is of the utmost importance to understand obesity and its association with the complex genetic architecture of the region.

**TABLE 1:** Latin American frequencies of SNPs associated with obesity, including South and Native American.

Gene	SNP	Frequency of reference allele			Frequency of alternative allele			Association
		ALFA Latin American	PAGE Study South American	PAGE Study Native American	ALFA Latin American	PAGE Study South American	PAGE Study Native American	
ADRB2	rs1042713	***G* = 0.55**	***G* = 0.52**	***G* = 0.54**	*A* = 0.44	*A* = 0.48	*A* = 0.46	Higher cholesterol levels
ADRB3	rs4994	*T* = 0.86	*T* = 0.83	*T* = 0.88	***C* = 0.14**	***C* = 0.17**	***C* = 0.12**	Increased levels of insulin, leptin, glucose, and lipids
APOA5	rs964184	***G* = 0.25**	***G* = 0.34**	***G* = 0.20**	*C* = 0.75	*C* = 0.66	*C* = 0.80	Lower HDL and higher triglyceride levels
	rs662799	***C* = 0.14**	***C* = 0.18**	***C* = 0.09**	*T* = 0.85	*T* = 0.82	*T* = 0.90	Higher levels of total cholesterol
CETP	rs3764261	***C* = 0.66**	***C* = 0.66**	***C* = 0.65**	*A* = 0.33	*A*−0.33	*A* = 0.35	Lower HDL
CYP1A2	rs762551	***C* = 0.33**	***C* = 0.22**	***C* = 0.30**	*A* = 0.67	*A* = 0.78	*A* = 0.70	Higher caffeine consumption
CYP2R1	rs10741657	*A* = 0.36	NA	NA	***G* = 0.64**	NA	NA	Vitamin D deficiency
CYP7A1	rs3808607	*C* = 0.35	*C* = 0.30	*C* = 0.42	***A* = 0.65**	***A* = 0.70**	***A* = 0.58**	Cholesterol levels
FABP2	rs1799883	***T* = 0.26**	***T* = 0.27**	***T* = 0.26**	*C* = 0.74	*C* = 0.73	*C* = 0.74	Fatty acid uptake
FADS1	rs174545	*C* = 0.49	*C* = 0.39	*C* = 0.61	***G* = 0.51**	***G* = 0.61**	***G* = 0.39**	Fatty acid metabolism
	rs174561	*T* = 0.51	NA	NA	***C* = 0.49**	NA	NA	
FADS2	rs174583	*C* = 0.48	*C* = 0.36	*C* = 0.57	***T* = 0.52**	***T* = 0.64**	***T* = 0.43**	Triglyceride levels
FTO	rs8050136	*C* = 0.67	*C* = 0.76	*C* = 0.65	***A* = 0.33**	***A* = 0.23**	***A* = 0.35**	Obesity predisposition
	rs9939609	*T* = 0.66	*T* = 0.76	*T* = 0.64	***A* = 0.34**	***A* = 0.24**	***A* = 0.34**	
FUT2	rs601338	***G* = 0.64**	NA	NA	*A* = 0.36	NA	NA	Vitamin B12 levels
GC	rs2282679	*A* = 0.77	NA	NA	***C* = 0.23**	NA	NA	Vitamin D deficiency
GIPR	rs2287019	***C* = 0.85**	***C* = 0.89**	***C* = 0.83**	*T* = 0.15	*T* = 0.11	*T* = 0.17	Obesity predisposition
IRS1	rs1522813	*G* = 0.68	NA	NA	***A* = 0.32**	NA	NA	Increased levels of glucose
LEP	rs7799039	*G* = 0.69	NA	NA	***A* = 0.31**	NA	NA	Food intake regulation
LIPC	rs1800588	*C* = 0.60	*C* = 0.48	*C* = 0.63	***T* = 0.40**	***T* = 0.52**	***T* = 0.37**	Higher LDL levels
MC4R	rs17782313	*T* = 0.82	*T* = 0.88	*T* = 0.81	***C* = 0.18**	***C* = 0.12**	***C* = 0.19**	Energy homeostasis and appetite regulation
	rs11872992	*G* = 0.86	*G* = 0.84	*G* = 0.88	***A* = 0.14**	***A* = 0.16**	***A* = 0.12**	
	rs8093815	*C* = 0.68	NA	NA	***T* = 0.32**	NA	NA	
	rs17066856	*T* = 0.82	NA	NA	***C* = 0.18**	NA	NA	
	rs1943218	*T* = 0.70	NA	NA	***C* = 0.30**	NA	NA	
	rs17066829	*T* = 0.65	NA	NA	***A* = 0.35**	NA	NA	
	rs9966412	*G* = 0.79	NA	NA	***A* = 0.21**	NA	NA	
	rs17066859	*G* = 0.87	NA	NA	***A* = 0.13**	NA	NA	
MYRF	rs174537	*G* = 0.61	*G* = 0.39	*G* = 0.61	***T* = 0.39**	***T* = 0.61**	***T* = 0.39**	LDL and cholesterol levels
MTHFR	rs1801133	*C* = 0.63	*C* = 0.58	*C* = 0.71	***T* = 0.37**	***T* = 0.42**	***T* = 0.29**	Folate deficiency
NOS3	Rs1799983	***T* = 0.24**	NA	NA	*G* = 0.76	NA	NA	Obesity predisposition
NPY	rs16147	***T* = 0.40**	***T* = 0.32**	***T* = 0.44**	*C* = 0.60	*C* = 0.68	*C* = 0.56	Higher obesity risk
PCSK1	rs6234	*G* = 0.76	*G* = 0.79	*G* = 0.75	***C* = 0.24**	***C* = 0.21**	***C* = 0.56**	Obesity predisposition
	rs236918	*C* = 0.93	*C* = 0.77	*C* = 0.82	***G* = 0.07**	***G* = 0.23**	***G* = 0.18**	Lower HDL levels
PPARG	rs1801282	*C* = 0.96	*C* = 0.89	*C* = 0.88	***G* = 0.04**	***G* = 0.11**	***G* = 0.12**	Obesity predisposition
TCF7L2	rs7903146	*C* = 0.72	*C* = 0.81	*C* = 0.74	***T* = 0.28**	***T* = 0.19**	***T* = 0.26**	Increased diabetes risk
TFR2	rs7385804	***C* = 0.31**	***C* = 0.25**	***C* = 0.34**	*A* = 0.69	*A* = 0.75	*A* = 0.66	Iron deficiency
UCP1	rs1800592	*A* = 0.60	*A* = 0.52	*A* = 0.65	***G* = 0.40**	***G* = 0.48**	***G* = 0.35**	Obesity predisposition
UGT1A1	rs6742078	*G* = 0.69	*G* = 0.63	*G* = 0.65	***T* = 0.31**	***T* = 0.37**	***T* = 0.35**	Bilirubin serum level

The obesity-associated risk alleles are in in bold.

### Single nucleotide polymorphisms associated with obesity and the frequencies in Latin America

*ADRB2* is a protein-coding gene that encodes a beta-2-adrenergic receptor, a member of the G protein-coupled receptor superfamily (22). The receptor’s primary function is the catecholamine-induced activation of adenylate cyclase (23). Moreover, The GO: Biological Process in which *ADRB2* is involved encompasses regulation of systemic arterial blood pressure by norepinephrine-epinephrine, diet-induced thermogenesis, G protein-coupled receptor signaling pathway, positive regulation of MAPK cascade, and adenylate cyclase-activating adrenergic receptor signaling pathway (24).

Furthermore, SNPs on *ADRB2* have been reported to be related to obesity. For example, reports regarding the SNP rs1042713 have described that carriers of the G allele were associated with higher insulin resistance, total cholesterol/high-density lipoprotein (HDL) ratio, total cholesterol, and low-density lipoprotein (LDL) than individuals with an AA genotype (25). The presence of the G allele in Latin America is higher, with an overall of 0.54, whereas the A allele is 0.46 (20, 21). Hence, the Latin American population may have an obesity predisposition and should avoid high-cholesterol foods.

Similarly, *ADRB3* is a protein-coding gene that modulates the catecholamine-induced activation of adenylate cyclase by the action of G proteins (22). The protein transcribed is part of the beta-adrenergic receptors family and is involved in lipolysis and thermogenesis regulation (23). Obesity has been associated with SNPs in this gene (22). The GO: Biological processes include diet-induced thermogenesis, eating behavior, positive regulation of cold-induced thermogenesis, generation of precursor metabolites and energy, norepinephrine-epinephrine-mediated vasodilation involved in the regulation of systemic arterial blood pressure, and carbohydrate metabolic process (24).

Moreover, an SNP (rs4994) in the *ADRB3* gene has been associated with obesity in different populations. For example, Xie et al. found an increased risk of childhood and adolescent obesity in individuals carrying the C allele in the East Asia population (26). Similarly, Daghestani et al. reported an association between rs4994 and the development of obesity and increased levels of insulin, leptin, glucose, and lipids in a Saudi population (27). The presence of the C allele in Latin America represents a 0.14, whereas the T allele is 0.86 (20, 21). Further studies are needed to determine the role of the SNP in the Latin American population by performing case-control studies.

*APOA5* is a protein-coding gene that encodes an apolipoprotein with a vital role in plasma triglyceride level regulation (22). The GO: Biological process encompasses the triglyceride metabolic process, lipid transport, cholesterol biosynthetic process, triglyceride catabolic process, regulation of intestinal cholesterol absorption, and triglyceride homeostasis (24). Mutations in *APOA5* have been correlated with hyperlipoproteinemia and hypertriglyceridemia (22).

Single nucleotide polymorphisms in *APOA5* have been associated with lower HDL and higher triglyceride levels (28–30). For instance, the SNP rs964184, in individuals with metabolic syndrome carrying the G allele, has been correlated with higher triglyceride levels and lower HDL in serum (28). Similarly, Qiu et al. found an association between the G allele in rs964184 and higher triglyceride concentration in Maonan and Han populations (29). In Latin America, the G allele has a frequency of 0.24, and the C allele, 0.76 (20, 21). Likewise, in the SNP rs662799, the allele C on the *APOA5* gene has been correlated with increased plasma lipids levels and higher total cholesterol levels (31). The frequency of the C allele in Latin America is 0.14, whereas, of the T allele, it is 0.86 (20, 21). Even though the frequencies of the associated SNP alleles are low, the Latin American population should be aware of the risk that consuming alcohol, fats, carbohydrates, and sugars has on human health (32).

*CETP* is a protein-coding gene located on chromosome 16. The protein encoded in this gene is involved in the transfer of neutral lipids, such as cholesteryl ester from HDL to other lipoproteins. Additionally, CETP regulates reverse cholesterol transport, sending the excess cholesterol to the liver for elimination (22, 23). Diseases associated with CETP encompass familial hyperlipidemia and hyperalphalipoproteinemia 1. The GO: Molecular functions of CETP are lipid, cholesterol, triglyceride, phosphatidylcholine binding, phospholipid transporter activity, and cholesterol transfer activity (24).

Moreover, an SNP (rs3764261) in the *CETP* has been associated with low HDL; individuals carrying the C allele have a strong risk factor for low HDL, which has been correlated with obesity (33, 34). In observational studies, significant interactions between diet and rs3764261 have been described. For example, in a study of 4,700 Iranian participants, a correlation between higher fish intake and a decrease in total cholesterol in individuals carrying the A allele was found (35). The frequency of the C allele in the Latin American population is 0.66, whereas for the A allele is 0.34 (20, 21). The C allele, associated with low HDL, is high in Latin America, increasing the obesity predisposition; hence, fish intake should be one of the principal components in the Latin American diet.

*CYP1A2* is a protein-coding gene located on chromosome 15. It encodes a cytochrome P450 monooxygenase, part of the cytochrome P450 superfamily of enzymes. These enzymes are involved in the metabolism of substrates such as steroids, cholesterol, and vitamins (22, 23). Reports have described the role of the CYP1A2 protein in the metabolism of carcinogenic intermediates from polycyclic aromatic hydrocarbons (PAHs) (22). Moreover, caffeine, aflatoxin B1, and acetaminophen have been reported as xenobiotics substrates of the enzyme (22). The GO: Biological process encompasses fatty acid, cholesterol, estrogen metabolic process, steroid catabolic and metabolic process, and cellular aromatic compound metabolic process (24).

An SNP (rs762551) in the *CYP1A2* gene has been reported to influence caffeine metabolism. For instance, studies have described that individuals carrying the A allele have a fast caffeine metabolism. On the other hand, the C allele was correlated with a slow metabolism and a higher risk of myocardial infarction (36, 37). Moreover, higher coffee consumption has been reported in rapid (A allele) compared to slow metabolizers (C allele), leading to a lower dietary fat intake (38). The frequency of the A allele in the Latin American population is 0.71, whereas, of the C allele, the frequency is 0.29 (20, 21). Latin America has a high coffee consumption, and based on those above; this could lead to a decrease in the fat intake in the region. However, further studies should be performed to understand the effect of caffeine on health.

Similarly, *CYP2R1* is a protein-coding gene located on chromosome 11. It encodes a cytochrome P450 monooxygenase, part of the cytochrome P450 superfamily of enzymes, which has an essential role in vitamin D precursors activation (22, 23). Additionally, CYP2R1 is involved in steroids, cholesterol, and other lipids synthesis. Diseases associated with this gene include rickets due to vitamin D deficiency (39). The GO: Biological processes include lipid, vitamin, and xenobiotic metabolic processes (24).

An SNP (rs1041657) in the *CYP2R1* gene has been correlated with vitamin D differences in serum. For instance, studies have reported that individuals carrying the G allele had lower vitamin D levels in serum, concluding that the rs1041657 has a vital role in determining vitamin D levels (40, 41). The frequency of the G allele in Latin America is 0.64, whereas for the A allele is 0.36 (20). Vitamin D levels have been associated with a preventive role in obesity, where individuals with low levels had a higher obesity predisposition (42). Based on this SNP frequency and vitamin D role, the diet in Latin America should aim for a high vitamin D intake.

Another member of the cytochrome P450 superfamily of enzymes is CYP7A1. The protein is a cytochrome P450 monooxygenase that has a crucial role in the metabolism of steroids, cholesterol, and other lipids. Moreover, the protein catalyzes the initial reaction of the cholesterol catabolic pathway in the synthesis of bile acids (22, 23). Hypercholesterolemia has been correlated with *CYP7A1* (22). The GO: Biological process of this gene encompasses lipid, steroid, sterol, cholesterol metabolic process, bile acid biosynthetic process, cholesterol catabolic process, response to organic cyclic compound, bile acid and bile salt transport, bile acid signaling pathway, cholesterol homeostasis, positive regulation of cholesterol biosynthetic process, and negative regulation of fatty acid biosynthetic process (24).

Furthermore, an SNP (rs3808607) in the *CYP7A1* gene has been correlated with serum cholesterol levels. For example, Iwanicki et al. found that individuals carrying the A allele in rs3808607 presented the highest total cholesterol concentration, whereas the C allele carriers had the lowest (43). The frequency of the A allele in the Latin American population is 0.65, while the C allele is 0.35 (20, 21). Thus, Latin American people may have a predisposition to high cholesterol, given the high frequency of the A allele. A balanced diet with low cholesterol intake should be followed.

*FABP2* is a protein-coding gene located on chromosome 4 (24). The protein encoded plays a vital role in the intracellular transport, metabolism, and uptake of fatty acids (FAs); moreover, FABP2 has a high affinity for binding saturated long-chain FAs (22, 23). The GO: Biological process includes fatty acid transport, metabolic process, and intestinal lipid absorption (24).

An SNP (rs1799883) in the *FABP2* gene has been associated with obesity. For example. Han, T-K and So, W-Y. described that carriers of the T allele had an increased incidence of central obesity and obesity-related metabolic syndrome in Korean women (44). Similarly, Kops et al. described that individuals carrying the T allele showed higher anthropometric profiles than individuals with the C allele (45). The frequency in Latin America of the T allele is 0.26, and for the C allele is 0.74 (20, 21).

*FADS1* is a protein-coding gene located on chromosome 11. The function of the protein encoded by *FADS1* is to introduce *cis* double bonds between carbons of the fatty acyl chain to regulate the unsaturation of FAs. It is also involved in the biosynthesis of highly unsaturated FAs and plays a role in the metabolism of inflammatory lipids (22, 23). Lipid metabolism disorder has been associated with *FADS1* (22). The GO: Biological process of FADS1 includes lipid, phospholipid, and fatty acid metabolic process, cell-cell signaling, cellular response to starvation, linoleic acid metabolic process, and regulation of cell differentiation (24).

Single nucleotide polymorphisms in *FADS1* have been correlated with fatty acid metabolism. For instance, carriers of the G allele in the rs174545 have been associated with higher triglyceride levels than C allele carriers (46). Mathias et al. described a significant association between rs174545 and alpha-linoleic, stearidonic, eicosanoic, and docosapentaenoic acids (47). The frequency of the C allele in Latin America is 0.49, and the G allele is 0.51 (20, 21). Similarly, the SNP rs174561 in the *FADS1* gene has been correlated with differences in polyunsaturated fatty acids (PUFA) ω-6 plasma concentrations in pregnant women, where carriers of the C allele showed higher concentrations of PUFA ω-6, in comparison with homozygotes with the TT genotype (48). In the Latin American population, the frequency of the C allele is 0.49, and for the T allele is 0.51 (20). Given the frequency of the risk alleles, the diet in Latin America should aim for a low fatty acid intake to prevent obesity.

The *FADS2* gene encodes a protein member of the fatty acid desaturase (FADS) family (24). The protein function includes the regulation of the unsaturation of FAs by introducing double bonds in the fatty acyl chain. Moreover, it has an important role in the biosynthesis of highly unsaturated FAs (22, 23). The GO: Biological processes include alpha-linolenic acid, lipid, fatty acid metabolic process, unsaturated, fatty acid biosynthetic process, and cellular biosynthetic process (24). Hyperlipoproteinemia has been associated with *FADS2* (22).

An SNP (rs174583) has been correlated with different triglyceride levels. For instance, Khodarahmi et al. reported significant differences in the atherogenic index of plasma and triglyceride levels. The carriers of the T allele had higher triglyceride concentrations than the carriers of the C allele (49). Similarly, Mazoochian et al. described increased triglyceride levels in individuals with the TT genotype (50). The T allele frequency in Latin America is 0.53, and the C allele is 0.47 (20, 21). Similar to *FADS1*, given the high frequency of the risk allele, a diet with low triglyceride levels should be followed in Latin America.

The *FTO* gene is located on chromosome 16. It encodes a nuclear protein involved in the demethylation of RNA. However, further studies are required to identify the specific physiological function. Glucose/energy metabolism is one of the FTO-related pathways (22, 23). FTO plays a role in the regulation of body fat accumulation and body size. Moreover, it is involved in adipocyte differentiation into brown or white fat cells (23). The GO: Biological processes include temperature homeostasis, DNA dealkylation involved in DNA repair, regulation of lipid storage, oxidative single-stranded DNA and RNA demethylation, adipose tissue development, regulation of white fat cell proliferation, and regulation of brown fat cell differentiation (24).

Single nucleotide polymorphisms in the *FTO* gene have been described as having a role in obesity. For example, Ahmad et al. reported that individuals carrying the A allele in the rs8050136 have a greater risk of obesity, higher BMI, and obesity-related conditions; however, by modifying the lifestyle and physical activity of the participants, the effect of the SNP was almost entirely blunted (51). The frequency of the A allele in Latin America is 0.31, and for the C allele is 0.69 (20, 21). Similarly, for the rs9939609, Sonestedt et al. reported that carriers of the A allele with low physical activity and a high-fat diet may be more predisposed to obesity (52). The frequency of the A allele in Latin America is 0.32, whereas, for the T allele is 0.68 (20, 21). Given the frequency of the risk alleles, the diet in Latin America should focus on low-fat intake, and daily physical activity.

*FUT2* is a protein-coding gene that encodes the galactoside 2-L-fucosyltransferase enzyme. The protein catalyzes the transfer of L-fucose to glycans chains on the cell surface; the resulting epitope is involved in different cellular processes, such as cell-cell interaction, and cell proliferation (22, 23). The gene is located on chromosome 19 and has been associated with differences in Vitamin B12 plasma levels (22). The GO: Biological processes include regulation of endothelial cell proliferation, carbohydrate and lipids metabolic process, protein glycosylation, and L-fucose catabolic process (24).

An SNP (rs601338) in the *FUT2* gene has been associated with vitamin B12 levels. The SNP rs601338 has been correlated with plasma vitamin B12 levels. Reports describe that individuals carrying the A allele have a higher vitamin B12 plasma concentration. This is in line with what has been found in different ethnic groups, correlating the frequency of the A allele with vitamin B12 plasma concentration. For instance, in the Indian population, the frequency of individuals carrying the G allele is higher than those carrying the A allele; hence, a lower vitamin B12 concentration is expected (53–56). The frequency of the A allele in Latin America is 0.36, and for the G allele is 0.64 (20). Reports have described low vitamin B12 concentrations in Latin America (57), which is in line with the frequency of the G allele in the region.

Moreover, studies have found an inverse correlation, associating low vitamin B12 levels with higher obesity predisposition (58). Thus, the Latin American population should increase their vitamin B12 intake to avoid an increased obesity risk. However, further studies should be performed to correlate the Latin American genotype with the vitamin B12 concentration.

*GC* is a protein-coding gene located on chromosome 4 (24). The protein encoded has a role in vitamin D binding and transport. Moreover, *GC* is part of the steroid and vitamin D metabolism pathways (22). The GO: Biological processes are vitamin D metabolic process, vitamin transport, and transmembrane transport (24).

Different SNPs in the *GC* gene have been associated with different vitamin D concentrations. For instance, Cheung et al. reported a correlation between the C allele in the SNP (rs2282679) and vitamin D deficiency in a Chinese population (59). Wang et al. reported that the rs2282679 was correlated with lower vitamin D levels (60). The frequency of the C allele in Latin America is 0.23, and of the A allele is 0.77 (20). Obesity and its genetic predisposition are complex; specific alleles could increase the obesity risk, and others may decrease it. Therefore, despite the genetic background, healthy food consumption should be the principal diet.

The *GIPR* gene encodes a G-protein coupled receptor for the gastric inhibitory polypeptide, which has been reported to stimulate insulin release. The GO: Biological processes encompass desensitization of the G protein-coupled receptor signaling pathway, generation of precursor metabolites and energy, signal transduction, G protein-coupled receptor signaling pathway, activation of adenylate cyclase activity, positive regulation of cytosolic calcium ion concentration, response to nutrients, calcium ions, FAs, and glucose (24).

The SNP rs2287019 has been associated with obesity and a higher BMI. For example, Speliotes et al. described a correlation between rs2287019 and an increased risk of developing obesity in individuals carrying the C allele (61). The frequency of the C allele in Latin America is 0.85, and the T allele is 0.15 (20, 21). The high frequency in Latin America is a matter of concern since obesity is now considered a significant health challenge in the region; hence, Latin America should aim to develop a healthier lifestyle to overcome this problem (4).

*IRS1* is a protein-coding gene located on chromosome 2. The protein encoded by this gene has been associated with the control of various cellular processes when phosphorylated by the insulin receptor tyrosine kinase. Type 2 diabetes has been correlated with IRS1 (22, 23). The GO: Biological processes encompass positive regulation of cell population proliferation, insulin receptor signaling pathway, positive regulation of glucose metabolic process, positive regulation of fatty acid beta-oxidation, cellular response to insulin stimulus, positive regulation of glycogen biosynthetic process, regulation of insulin receptor signaling pathway (24).

Single nucleotide polymorphisms in the *IRS1* gene have been associated with diabetes and higher glucose levels. For instance, He et al. reported an increased risk of developing diabetes in women carriers of the A allele of the SNP rs1522813, compared to carriers of the G allele (62). The frequency of the A allele in Latin America is 0.32, and of the G allele, 0.68 (20). Similarly, Ohshige et al. reported a significant association between the C allele and the risk of type 2 diabetes in a Japanese population (63); this is in line with what was reported in a European population (64). The frequency of the C allele in Latin America is 0.75 and of the T allele 0.25 (20, 21).

Moreover, diabetes has been associated with obesity in both ways, with obesity increasing the risk of diabetes, and vice versa (65). In diabetes, the glucose does not enter the cell; it remains in the blood, where it is converted into FAs, and stored as fat (65). The high frequency of the C allele in the Latin American population is alarming, so public policies should consider the genetic information of the region.

The *LEP* gene encodes a protein called leptin which plays a role in energy homeostasis regulation. The protein binds to its receptor in the hypothalamus, activating signaling pathways that promote energy consumption and inhibit feeding. The protein also regulates the secretion of hormones in the brain. Mutations in the *LEP* gene have been significantly associated with obesity. Diseases correlated to LEP include leptin deficiency and overnutrition (22, 23). The GO: Biological processes include regulation of protein phosphorylation, response to dietary excess, glucose metabolic process, energy reserve metabolic process, regulation of blood pressure, and adult feeding behavior (24).

An SNP (rs7799039) in the *LEP* gene has been associated with obesity. For instance, Zayani et al. found a significant association between obesity and carriers of the A allele (66). This agrees with what Bains et al. found in an Indian population (67). The frequency of the G allele in Latin America is 0.69, and for the A allele is 0.31 (20). Overeating is one of the principal causes of obesity; however, the origin of overeating may be in the person’s genetic information (68). Based on the high frequency of the A allele, obesity in Latin America could be predisposed by SNPs in the *LEP* gene. Thus, genetic studies should be part of the diagnosis and obesity treatment.

The *LIPC* gene encodes a hepatic triglyceride lipase, which catalyzes phospholipid and triglyceride hydrolysis. The protein is involved in the triacylglycerol biosynthesis pathway (22, 23). The GO: Biological processes include lipid and cholesterol metabolic process, fatty acid biosynthetic process, cholesterol transport, lipoprotein particle remodeling, and cholesterol and triglyceride homeostasis (24).

An SNP (rs1800588) in the *LIPC* gene has been associated with LDL cholesterol and triglyceride levels. Carriers of the T allele had higher LDL cholesterol and triglyceride levels than carriers of the C allele (69). The frequency of the C allele in the Latin American population is 0.57, and for the T allele, 0.43 (20, 21). Based on the frequency of the SNP, the diet in Latin America should be focused on a low LDL and triglycerides intake.

The *MC4R* gene is located on chromosome 18 and encodes a membrane-bound receptor, part of the melanocortin receptor family. The protein plays a crucial role in somatic growth and energy homeostasis. Moreover, mutations causing a deficiency in the MC4R protein have been associated with obesity (22, 24). The GO: Biological processes encompass diet-induced thermogenesis, energy reserve metabolic process, G protein-coupled receptor signaling pathway, feeding behavior, regulation of the metabolic process, and regulation of eating behavior (24).

Several SNPs in *MC4R* have been correlated with obesity since the protein encoded by it plays a vital role in weight control, energy balance, and food intake. Thus, loss-of-function mutations will disrupt the pathways where MC4R is involved (70). For example, Yu et al. reported an association between the C allele in the rs17782313 and a higher risk of developing obesity (71). The frequency of the C allele in Latin America is 0.17, and for the T allele is 0.83 (20, 21). Moreover, more SNPs in the *MC4R* have been associated with alterations in energy balance and food intake, including rs11872992, rs8093815, rs17066856, rs1943218, rs17066829, rs9966412, and the rs17066859 (61, 70–73). On the other hand, gain-of-function mutations have been negatively correlated with obesity (72).

The gene *MTHFR* encodes a protein that catalyzes the conversion of 5,10-methylenetetrahydrofolate to 5-methyltetrahydrofolate. Moreover, it has been highly associated with folate (22, 23). Among its related pathways, the metabolism of glucose is included. Mutations in this gene have been associated with homocystinuria, and neural tube defects (22). The GO: Biological processes correlated with *MTHFR* include neural tube closure, metabolic process, response to vitamin B2, tetrahydrofolate interconversion, and response to folic acid (24).

Single nucleotide polymorphisms in the gene *MTHFR* have been associated with folate deficiency (74, 75). For example, for the SNP rs1801133, individuals with the genotype CT had an enzyme activity of 67%, and individuals with the TT genotype had 25% activity (74). Similarly, reports have found that carriers of the T allele have lower folate levels (76). Moreover, folate deficiency has been identified as an obesity risk factor (77). The frequency of the C allele in Latin America is 0.64, whereas the T allele is 0.36 (20, 21). Based on the allele frequency of the SNP and the association of folate with obesity, an appropriate folate intake is vital for maintaining good health.

The gene *MYRF* encodes a transcription factor involved in the myelination of the central nervous system. The protein promotes the expression of myelin production genes (22, 23). The GO: Biological processes associated with *MYRF* include regulation of transcription, proteolysis, oligodendrocyte development, and central nervous system myelination. Moreover, SNPs in this gene have been associated with obesity (24).

An SNP (rs174537) in the *MYRF* gene has been correlated with different levels of LDL and cholesterol. For example, Tanaka et al. described that carriers of the G allele had higher LDL and cholesterol levels in serum than individuals with the T allele (78). The frequency of the G allele in Latin America is 0.50, the same for the T allele (20, 21). Given the high frequency of the risk allele in the Latin American population, a diet with low LDL and cholesterol should be followed.

The *NOS3* gene is a protein-coding gene located on chromosome 7. The protein encoded is a nitric oxide synthase 3 involved as a biological mediator in different processes. L-arginine is used as a precursor for nitric oxide synthesis (22). Nitric oxide is related to vascular smooth muscle relaxation, angiogenesis, and blood clotting. Moreover, diseases correlated with *NOS3* are Alzheimer’s, stroke, and obesity (22, 23, 79).

The SNP (rs1799983) in the *NOS3* gene has been associated with obesity. For instance, Nasr et al. described an association between rs1799983 and obesity, individuals carrying the T allele had a higher obesity predisposition than carriers of the G allele (79). Similarly, Pawlik et al. found a correlation between the T allele and higher obesity-related parameters, such as a higher BMI (80). The frequency of the T allele in Latin America is 0.24, whereas the G allele is 0.76. Hence, regarding the *NOS3* gene, only a small proportion of the Latin American population may be predisposed to obesity. However, maintaining a balanced diet is crucial to avoid obesity.

The gene *NPY* encodes a neuropeptide associated with several physiological processes, such as circadian rhythms, cardiovascular function, stress response, and food intake (22). The GO: Biological processes of the gene include the neuropeptide signaling pathway, chemical synaptic transmission, feeding behavior, positive regulation of appetite, dopamine metabolic process, and eating behavior. Diseases associated with the *NPY* gene are eating disorders (24).

Moreover, SNPs in the *NPY* have been associated with obesity. For example, Zain et al. described that carriers of the T allele in the SNP (rs16147) were correlated with high obesity parameters such as BMI, triglyceride levels, and body fat percentage, and increased obesity risk in comparison with carriers of the C allele (81). Lin et al. reported similar obesity-associated findings; however, the effects of rs16147 were blunted by a low-fat diet (82). The frequency of the T allele in Latin America is 0.40, whereas, of the C allele, the frequency is 0.60 (20, 21). Based on the high frequency of the T allele, the diet in Latin America should be focused on a low-fat intake.

The gene *PCSK1* is a protein-coding gene located on chromosome 5. The protein encoded by *PCSK1* is part of the subtilisin-like proprotein convertase family, which is involved in the protein and peptide precursors trafficking. Moreover, the protein participates in the activation of neuropeptide precursors and polypeptide hormones (22). Mutations in *PCSK1* have been associated with obesity predisposition (39).

Single nucleotide polymorphisms in the *PCSK1* gene have been correlated with obesity predisposition. For instance, Benzinou et al. described an association between the presence of the C allele in rs6234 and an increased risk of developing severe obesity in a European population (83). Similarly, Nead et al. found that the C allele not only predisposes the carrier to severe obesity but increases the risk of common obesity and higher BMI (84); with a frequency of the C allele in Latin America of 0.23, and for the G allele, 0.77 (20, 21).

*PPARG* is a protein-coding gene located on chromosome 3. The protein encoded by *PPARG* is a nuclear receptor, part of the peroxisome proliferator-activated receptor subfamily of nuclear receptors. After ligand binding, the protein modulates the peroxisomal beta-oxidation pathway of FAs, glucose homeostasis, and adipocyte differentiation (22, 23). Mutations in the *PPARG* gene have been associated with diseases such as atherosclerosis, diabetes, and obesity (22, 39).

An SNP in *PPARG* has been correlated with obesity. Li et al. described an association between the G allele in the SNP rs1801282 and higher levels of BMI, and total cholesterol, increasing obesity predisposition and hypercholesterolemia, in comparison with CC homozygotes (85). The frequency of the C allele in Latin America is 0.92, and for the C allele is 0.08 (20, 21). Although the risk allele frequency is low, a balanced diet should be followed for a healthy lifestyle.

The *TCF7L2* gene encodes a transcription factor that participates in the Wnt signaling pathway, regulating MYC expression by repressing or activating its transcription. Mutations in the *TCF7L2* gene have been associated with increased blood glucose levels, and diabetes (22, 39). The GO: Biological processes associated include the Wnt signaling pathway, positive regulation of insulin secretion, glucose homeostasis, canonical Wnt signaling pathway, and fat cell differentiation (24).

Furthermore, SNPs in the *TCF7L2* gene have been associated with obesity and diabetes. For example. Wrzosek et al. found that the T allele in the rs7903146 was associated with an increased risk of developing diabetes compared to carriers of the C allele (86). Similarly, Yazdi et al. described a significant association between the T allele in the rs7903146 and type 2 diabetes (87). The frequency of the C allele in Latin America is 0.76, and of the T allele is 0.24 (20, 21). Even though the risk allele frequency is not high in the region, the diet should be focused on a healthy intake of micro and macronutrients.

*TFR2* is a protein-coding gene located on chromosome 7. The protein encoded by *TFR2* is a single-pass type II transmembrane protein, part of the transferrin receptor-like family. The protein participates in the cellular uptake of transferrin-bound iron. Hemochromatosis is a disease associated with *TFR2* (22, 23). The GO: Biological processes include iron ion transport and homeostasis, transferrin transport, cellular response to iron ions, and positive regulation of peptide hormone secretion (24).

Moreover, SNPs in *TFR2* have been associated with dysregulations in iron levels. For instance, An et al. found that carriers of the C allele in the SNP rs7385804 correlated with lower serum ion levels, compared to A allele carriers (88). Furthermore, low iron levels have been associated with obesity and anemia (89). The frequency of the C allele in Latin America is 0.3, while of the A allele is 0.7 (20, 21). In Latin America, iron deficiency is a problem; hence, the diet should be focused on reaching the iron requirements and avoiding health issues, such as anemia (90).

The gene *UCP1* is a protein-coding gene located on chromosome 6. The protein encoded is part of the mitochondrial uncoupling proteins, which are involved in separating oxidative phosphorylation from ATP synthesis, producing heat in this process (22, 23). Moreover, the protein participates in the reduction of the mitochondrial membrane potential in mammalian cells. The *UCP1* gene is only expressed in brown adipose tissue, specialized in heat production (22, 23). The GO: Biological processes associated include diet-induced thermogenesis, mitochondrial transport, response to temperature stimulus, response to nutrient levels, brown fat cell differentiation, and adaptive thermogenesis (24).

Furthermore, SNPs in the *UCP1* gene have been associated with obesity. For instance, carriers of the G allele in the SNP rs1800592 have been correlated with an increased risk of developing moderate obesity in comparison with the A allele. Thus, the SNP could play a role in the initial stages of obesity (91). The frequency of the G allele in the Latin American population is 0.41, whereas the A allele is 0.59 (20, 21). The high frequency of the risk allele in Latin America could be associated with the increasing obesity tendency in the region (4); hence the diet should include an adequate caloric intake. Further studies should be performed in Latin America to understand the extent of the SNP in obesity.

*UGT1A1* is a protein-coding gene located on chromosome 2. The protein encoded by *UGT1A1* is a UDP-glucuronosyltransferase, which participates in the transformation of lipophilic molecules, such as hormones, steroids, and bilirubin, into excretable metabolites. However, the enzyme has a higher affinity for bilirubin (22). UGT1A1 is involved in the elimination of endogenous compounds, xenobiotics, and drugs (23). Diseases associated with *UGT1A1* include Gilbert syndrome and dysregulation of bilirubin (22, 39).

Moreover, SNPs in the *UGT1A1* gene have been associated with different bilirubin levels. For example, Abbasi et al. described a correlation between the T allele in the SNP rs6742078 and higher bilirubin levels, in comparison with the G allele (92). Moreover, high bilirubin levels have been related to a higher risk of developing diabetes (93). The frequency of the G allele in Latin America is 0.66, whereas the T allele is 0.34 (20, 21). Thus, the diet in Latin America should include foods that could reduce the bilirubin levels, such as flavonoid-rich fruits and vegetables (94).

## Genomics and diet interaction in obesity in the Latin American environment

The application of omics sciences, such as genomics, has facilitated the study of genetic variants in diseases such as obesity. Genetic and environmental factors contribute to obesity. Therefore, it is crucial to analyze the interaction between factors such as gene expression and food intake to implement dietary guidelines. These diets could modify gene expression at the pre-, post-transcriptional, or translational level and reduce the risk of developing diseases (12, 15).

The nutrition effects on metabolic pathways or oxidative and inflammatory stress are the subject of several nutrigenomic investigations worldwide. Carbohydrates, proteins, fats, and vitamins are essential for body’s normal function. Various genes have been associated with the absorption of nutrients (95).

Ignatieva et al. designed a compendium comprising 578 human genes controlling feeding behavior and body weight. This compilation included genes from the scientific literature, such as *ADRB2*, *LEP*, *NPY*, *PCSK1*, *PPARG*, *UCP1*, *APOA5*, *IRS1*, and others. Moreover, they found that genes from the compendium list were expressed in seven tissues or organs: adipose tissue, breast tissue, adrenal gland, pituitary gland, pancreas, liver, and whole brain. Each of the tissues and organs plays an essential role in metabolism; for example, the adrenal and pituitary glands control metabolism through humoral signals. Adipose tissue and the breast are related to lipid storage. The liver is essential for lipogenesis, gluconeogenesis, and cholesterol metabolism. Finally, the brain regulates eating behavior through sensory signals such as taste, smell, and texture of food. (95–97).

In this section, a bibliographical selection of several studies that examined the impact of diet on the gene expression of numerous genes involved in various metabolic pathways has been selected. The studies were related to obesity, weight loss, metabolism related to insulin resistance, and elevated lipid and carbohydrate levels.

Table 2 includes a list of dietary factors that influence gene expression of genes involved in various metabolic pathways. This information reveals different comparative approaches, such as (a) The difference between after (post) and before (pre) any intervention (bariatric surgery); (b) the variation between groups consuming distinct types of diet; (c) many studies were performed in small groups of individuals; (d) nine studies used animal models in their research; and (e) some of the studies were published decades ago.

**TABLE 2:** Influence of dietary factors in gene expression of genes associated with obesity.

Dietary factors	Target genes	Regulation up or down	Potential health effects	Type sample	References
Lipid metabolism and insulin	ADRB2	Down	The expression of ADRB2 was significantly lower in the adipose tissues of obese patients than in tissues of normal-weight individuals	Adipose tissues	(98)
Lipid metabolism	ADRB3	Down	ADRB3 expression levels in adipocytes were downregulated before the onset of obesity, indicating that reduced ADRB3 expression might be the cause of obesity.	Mature adipocytes and adipose tissue stromal vascular cells	(136)
Triglyceride metabolism	APOA5	Down	The amount of apoA5 was significantly reduced by 69% in the obese group as compared with the non-obese group	Human subcutaneous abdominal adipose tissues	(137)
HDL metabolism	CETP	Up	HDL concentration and CETP expression are correlated; if HDL concentration is higher, CETP expression is also higher.	Subcutaneous abdominal adipose tissue	(138)
Xenobiotic metabolism	CYP1A2	Down	The high-fat diet curtailed the activity and the expression the CYP1A2 in obese male and female mice.	Liver tissue	(139)
Vitamin D deficiency	CYP2R1	Down	Obesity represses CYP2R1 expression in human adipose tissue.	Adipose tissue	(110)
Cholesterol metabolism	CYP7A1	Up	Relative to the high fat diet fed group, the low fat diet fed animals showed reductions in the hepatic expression of CYP7A1.	Hepatic tissue	(140)
Free fatty acid metabolism	FABP2	Down	FABP2 expression was intermediate in the duodenum, highest in the ileum, and close to zero in the colon.	Duodenal, ileum, and colon biopsy samples	(132)
Lipid metabolism	FADS1	Down	FADS1 gene expression was lower in duodenum and jejunum 3 months following Roux-en-Y gastric bypass, compared to before surgery.	Mucosa from stomach, duodenum, jejunum and ileum	(141)
Lipid metabolism	FADS2	Down	FADS1 and FADS2 mRNA levels were significantly reduced in the TT carriers compared with the CC and TT carriers.	Visceral adipose tissues	(142)
Glucose and lipid metabolism	FTO	Up	The relative gene expressions in overweight/obese were significantly decreased at the end of Ramadan intermittent fasting.	Whole blood sample	(121)
Lipid metabolism and vitamin B12 levels	FUT2	Up	Fut2 mRNA had significantly lower expression after Western diet feeding for 20 weeks in an obese mouse model.	Ileum and colon tissue	(143)
Glucose and fat metabolism	GIPR	Down	GIPR expression was downregulated in human adipose tissue from obese patients and correlated negatively with body mass index, waist circumference, systolic blood pressure, and glucose and triglyceride levels.	Human sc adipose tissue	(144)
Insuline resistance	IRS1	Down	The reduced expression of IRS-1 in visceral adipose tissue of morbidly obese people suggests that IRS-1 expression plays a prognostic role in visceral adipose tissue’s insulin responsiveness.	Visceral adipose tissue	(145)
Glucose metabolism	LEP	Up	An increased expression of LEP was detected in the subcutaneous fat of the obese group compared to control.	Subcutaneous fat tissue	(146)
Lipid metabolism	LIPC	Down	High-fat diet feeding significantly decreases hepatic lipase activity (LIPC) in mice	Liver tissue	(147)
Appetite regulation	MC4R	Down	They found a significant decrease in MC4R mRNA expression in rats fed a high-fat diet compared to expression levels in rats fed a normal diet.	Adipose tissue	(148)
Folate metabolism	MTHFR	Up	MTHFR expression was directly correlated with severe obesity.	Visceral adipose tissue	(149)
Omega 3	MYRF	Up	Several genes were associated with the progression of obesity-associated type 2 diabetes mellitus. Inconclusive results for MYRF.	GEO database: GPL20301 dataset	(150)
Lipid metabolism and obesity	NOS3	Up	Increased gene expression of NOS3 may cause decreased lipolysis of subcutaneous adipose tissue in obesity.	Adipose tissue.	(151)
Appetite regulation	NPY	Up	NPY overexpression in the paraventricular nucleus causes obesity by increasing food intake, whereas NPY knockdown in the hypothalamus promotes energy expenditure.	Hypothalamus rats	(152)
Lipid and cholesterol metabolism appetite regulation	PCSK1	Down	Inconclusive study	Pancreatic tissue of mice	(153)
Lipid and carbohydrates metabolism	PPARG	Up	PPARG mRNA expression is most abundant in serum of obese patients both diabetic and non-diabetic.	Serum	(154)
Insuline resistance	TCF7L2	Down	Obesity was associated with reduced TCF7L2 transcript levels in whole subcutaneous abdominal AT but paradoxically increased expression in adipose progenitor cells.	Subcutaneous abdominal adipose tissue	(155)
Iron levels	TFR2	Up	Increased tfR2 expression and the presence of iron.	Adipose tissues from obese mice	(156)
Glucose and energy balance	UCP1	Up	UCP1 mRNA expression in had significant negative correlations with obesity-related markers.	Abdominal visceral adipose tissue	(157)
Non-alcoholic fatty liver disease Bilirubin levels	UGT1A1	Up	These data demonstrated obesity- and fasting-induced UGT1A1 increased expression in mouse liver.	Liver tissue	(158)

### Influence of ADBR2 and ADBR3 on thermogenesis

Obesity is characterized by a long-term imbalance in energy homeostasis, which is influenced by adaptive thermogenesis. The sympathetic nervous system regulates thermogenesis and is produced in muscle and brown adipose tissue. Adipose tissue contains adrenergic receptors like the beta-3 adrenergic receptor (*ADRB3*) and the beta-2 adrenergic receptor (*ADRB2*). Both *ADRB2* and *ADRB3* are receptors for thermogenesis and lipolysis regulation, which provides free FAs for thermogenesis (98).

Defective expression of ADRBs on the cell surface or their altered signaling can result in decreased lipolysis and thermogenesis, which may contribute to obesity. *ADRB3* is found primarily on the surface of visceral and brown adipose cells and stimulates sympathetic nerves to release noradrenaline in response to cold temperatures or food consumption. *ADRB2* regulates catecholamine function and may be important in obesity because catecholamines contribute to energy expenditure and lipolysis (99). Research in mice reported that *ADRB2* is also expressed in hypothalamic neurons, confirming its role in the central regulation of eating behavior (100).

### CYP1A2

Morbid obesity and changes in body weight and composition are parameters that can influence cytochrome P450 (CYP) superfamily activities and, consequently, drug metabolism. The liver is the main organ responsible for the metabolization and detoxification of xenobiotic molecules, such as caffeine, exogenous toxins, and drugs. The CYP family are drug heme-metabolizing enzymes and play an essential role in protecting the body against both endogenous and exogenous toxic compounds. These enzymes are involved in the metabolism of drugs and phase I toxins, which contributes to the target compounds being more hydrophilic and more easily excreted in the bile or urine. In addition, the dietary composition can alter the expression and activity of many CYP proteins, influencing drug metabolism and disease prevalence. CYP1A2 is one such enzyme, and it is responsible for about 5% of drug metabolism in humans.

### Vitamin D deficiency related to *CYP2R1* and *GC*

Certain studies indicate that obesity-related disorders or excess body fat could be associated with vitamin D deficiency (101). Vitamin D is an active prohormone necessary for bone tissue maintenance and calcium and phosphorus homeostasis. Ergocalciferol, often known as vitamin D2, and cholecalciferol, known as vitamin D3, are the two natural forms of vitamin D. D3 is derived from a diet rich in oily fish, liver, egg yolk, and fortified foods such milk, bread, and margarine. In contrast, D2 comes from the conversion of ergosterol, a plant sterol obtained from a diet that includes only plant foods such as yeast and mushrooms (102).

Vitamin D (both D2 and D3 forms) from food is absorbed by bile salts action in the distal part of the small intestine and then transported by vitamin D–Binding Protein (GC), albumin, or LDL to different tissues and organs. When vitamin D enters the liver, it undergoes its first hydroxylation at carbon-25 *via* the enzyme 25-hydroxylase (CYP2R1), making 25-hydroxyvitamin D [25(OH)D], or calcidiol that is biologically inactive (103). The 25(OH)D needs to undergo a second hydroxylation at carbon-1 by the enzyme 1α-hydroxylase (CYB27B1), which is mainly found in the kidneys and produces 1,25-dihydroxy vitamin D [1,25(OH)2D] or calcitriol, to become active. Finally, calcitriol binds to the nuclear vitamin D receptor and regulates calcium homeostasis and bone metabolism (104). This has led to the routine use of measuring the plasma concentration of 25(OH)D to identify people at risk of vitamin D insufficiency.

At the level of Latin America, vitamin D deficiency was classified as a mild, moderate, or severe public health problem, depending on the subgroups evaluated in each country. For example, in Mexico, a 10% prevalence of vitamin D insufficiency was found (25 -hydroxyvitamin D < 50 nmol/L) in adults (102). In addition, a study evaluated the diet in different regions of Latin America, currently obtaining a diet pattern based on total fats, an increase in animal products, and a decrease in the consumption of cereals, fruits, and some vegetables (105). In this sense, Sharifan et al. suggested that a diet characterized by high consumption of fruits, green leafy vegetables, honey, dairy products, olive oil, nuts, legumes, and low consumption of sugar and solid fats was associated with better serum concentrations of 25(OH)D (106). Consequently, the diet could influence the regulation of vitamin D bioavailability. Nevertheless, other studies report that the deficiency of this vitamin is attributed to the lack of exposure to the sun, especially in obese people who, due to their weight, limit their movement and prefer to cover their bodies (107).

Research has shown that obesity suppresses CYP2R1 expression. Studies in mice showed that obesity inhibited the expression of CYP2R1 in mouse livers, which was linked to a decrease in enzyme 25-hydroxylation activity, influencing fluctuations in the levels of 25-OH-D in the blood (108, 109). It shows that decreased vitamin D hydroxylation could play a role in obesity-induced vitamin D deficiency. Another study analyzed CYP2R1 expression from abdominal adipose tissue samples from four female patients who underwent gastric bypass surgery, suggesting that obesity represses CYP2R1 expression in human adipose tissue and that weight loss restores CYP2R1 (110). Decreased CYP2R1 expression could be due to high fat intake affecting the amount of vitamin D absorbed from food. Several studies have reported that vitamin D metabolites can be retained by excess body fat. Likewise, cholecalciferol can be largely sequestered by body fat before being transported to the liver due to its reduced hydrophobicity (107).

Another critical factor affecting 25-OH-D levels is vitamin D binding protein (VDBP) and the GC gene codes for VDBP. The lower plasma concentrations of 25-OH-D may be due to decreased hepatic synthesis of VDBP. In one study they found that, unlike CYP2R1, there is insufficient evidence that obesity influences VDBP expression in mouse liver, suggesting that these two crucial indicators of vitamin D status are controlled differently (110).

### *CYP7A1* and cholesterol

Cholesterol is a structural component of cell membranes and a precursor of steroid hormones and bile acids. Cholesterol is converted to bile acids in the liver, removing it from the active cholesterol pool and leading to an increase in hepatic LDL receptors and a decrease in plasma cholesterol levels. Bile acids are also excreted into the small intestine, where they act as detergents to aid in the absorption of dietary cholesterol, lipids, and fat-soluble vitamins. Bile acids are reabsorbed in the distal ileum and returned to the liver, but only once per cycle. Thus, cholesterol removal from the body is facilitated by conversion to bile acids, which may also influence plasma cholesterol levels (111).

Dietary cholesterol regulates the expression of many genes in the liver. Cholesterol 7-hydroxylase (Cyp7a1) is a candidate gene for this function. CYP7A1 is a rate-limiting enzyme in the bile acid synthesis pathway; bile acids influence energy expenditure and glucose and lipid metabolism. CYP7A1 is found in the endoplasmic reticulum (ER) of hepatocytes (112).

The research evaluated the effects of diets with 0.0 and 0.5% cholesterol in different mice. They showed that transgenic mice overexpressing Cyp7a1 in the liver were resistant to obesity, fatty liver, and high-fat diet-induced insulin resistance. These results suggest that Cyp7a1 regulation could be an important determinant of plasma cholesterol responsiveness (113).

### *FADS1* and *FADS2* are regulated by polyunsaturated fatty acids

Polyunsaturated fatty acids are widely acknowledged to have a significant impact on human health. PUFAs have been linked to various clinical outcomes, including obesity and metabolic syndrome. Fatty acid desaturase 1 (*FADS1*) and fatty acid desaturase 2 (*FADS2*) have been studied as candidate genes for endogenous conversion of 18-carbon PUFAs into very long-chain FAs such as arachidonic acid, docosahexaenoic acid, and eicosapentaenoic acid. The lipogenic transcription factors SREBP1c and peroxisome proliferator-activated receptors (PPARs) regulate gene expression of the FADS1 and FADS2, primarily in the liver but also in adipose tissue (114). Several studies have found that high-fat diets reduce the expression of Fads1 and Fads2 in a variety of hepatic models, ranging from human HepG2 cells treated with different PUFAs to mice and baboons. Furthermore, PUFA-mediated decreases in Fads expression are mirrored in liver fatty acid content (115, 116).

One study evaluated FADS1 and FADS2 expression in adipocytes treated with α-linolenic, linoleic, eicosapentaenoic, or arachidonic acid. They observed reductions in the expression of the FADS2 protein gene but not in FADS1. Concluding that these adipocytes have a functional FADS pathway that can be regulated by PUFA (117).

### Relationship of glycogenesis and lipogenesis with *FTO*

Alpha-ketoglutarate-dependent dioxygenase (FTO) is the gene with the most significant impact on obesity. FTO acts as a cellular sensor for some nutrients like lipids and glucose (118, 119). FTO regulates the expression of hepatic gluconeogenic genes such as G6PC (Glucose-6-phosphatase) and PCK (Phosphoenolpyruvate carboxykinase) by altering the activity and interaction with transcription factors such as STAT30 (Signal Transducers and Activators of Transcription 3), CREB (protein cAMP-responsive element binding), and ATF4 (activating transcription factor 4) (120). Increased FTO expression causes increased transcription of genes encoding gluconeogenic enzymes, leading to increased gluconeogenesis, while decreased FTO expression causes the opposite effect (121). For example, Doaei et al. report that the FTO expression level in peripheral blood mononuclear cells increased in obese individuals (122). Furthermore, FTO regulates hepatic lipid metabolism by changing the methylation status of genes involved in fatty acid oxidation, lipolysis, and *de novo* lipogenesis. Increased FTO expression decreases CPT1, LIPE, and ATGL mRNA expression, resulting in decreased fatty acid oxidation and lipolysis. It also raises ATF4 expression, which stimulates the expression of lipogenic genes, resulting in increased *de novo* lipogenesis in the liver (120).

### FUT2

Vitamin B12 is obtained from food or synthesized by microorganisms in the gut in humans. Three proteins in the body are responsible for its absorption, transport, and cellular uptake: haptocorrin, intrinsic factor, and Transcobalamin II (123). Vitamin B12 is essential for many processes, including the formation of red blood cells, DNA synthesis, and the maintenance of the myelin nerve sheath. Variations in the FUT2 gene may increase the risk of Helicobacter pylori (H. pylori) infection and the associated gastric-induced vitamin B12 malabsorption. Infections with H. pylori in the human intestine have been shown to inhibit the release of intrinsic factors, required for vitamin B12 absorption (124).

A study reported that the association between genetically low vitamin B12 concentrations and cardio-metabolic traits could be modified by dietary intake. They evaluated in a Brazilian population, the metabolism and concentration of vitamin B12. As a result, they showed a significant interaction between dietary carbohydrate and protein intake on LDL cholesterol and homocysteine concentrations in obese individuals (125).

### Leptin

Imbalanced expenditure of energy leads to excess body fat. The leptin (LEP) hormone plays a significant role in the energy balance and control of body weight. Levels high of leptin are associated with obesity. Furthermore, obese patients with insulin resistance have a higher concentration of circulating leptin than normal-weight people (126). Insulin resistance is facilitated by leptin, a negative regulator of insulin.

Leptin is also involved in other physiological processes, such as glucose metabolism. Thus, through activation of its LEPR receptor, leptin reaches numerous brainstem regions, including the hypothalamus, helping regulate glucose and energy balance. Another way to reverse the high glucose levels or increase insulin sensitivity is indirect hypoglycemia, where glucose binds to the liver’s leptin receptor to regulate hepatic glucose metabolism (109). When leptin is present, the body’s tissues can absorb more insulin and glucose. Therefore, if there are alterations in leptin receptor expression or inhibition of the activator of transcription-3 (*STAT-3*), the leptin signaling pathway will not be activated (127, 128).

Dietary factors, such as overeating, including fats or sugars, can generate molecular mechanisms that lead to leptin resistance (129). For example, dietary sugar and saturated fat elevate plasma triglycerides, and several animal and human studies demonstrate how particular macronutrient patterns correlate with circulating leptin levels. In a study of healthy women, an increase in carbohydrate intake (bread, rice, and sugar) led to plasma leptin levels increasing by 28% and an increase in energy expenditure of 7%. Therefore, fructose removal from high-fat diets can reverse leptin resistance (130).

### Proprotein convertase subtilisin/kexin type 1

Obesity is associated with changes in the melanocortin pathway, a crucial factor in energy homeostasis. The central melanocortin system regulates food intake and energy expenditure through pro-opiomelanocortin (POMC) neurons. POMC is cleaved sequentially by two prohormone convertases, PC1/3, and PC2, and processed by three enzymes to at least three melanocortin peptides. These peptides are essential melanocortins involved in the regulation of appetite and body weight (37).

Prohormone convertase 1/3 (PC1/3) is encoded by the proprotein convertase subtilisin/kexin type 1 (PCSK1) gene. Various human genetics studies have associated PCSK1 with metabolic phenotypes such as early onset obesity, intestinal malabsorption, gastrointestinal complications, diabetes, and reactive hypoglycemia (38, 39). The availability of various PCSK1 mouse models made it possible to investigate its function and expression in different tissues (brain, brainstem, pancreas, intestine, stomach, and immune cells) (40, 41).

### TCF7L2

*TCF7L2* encodes a protein that acts as a transcription factor and participates in the formation of pancreatic β-cells needed to reduce blood sugar (97). One study evaluated the association between TCFL2, obesity, and diabetes in adipose tissue, reporting that TCFL2 expression decreased in rats fed a 60% fat diet compared to a 10% fat diet. Based on these findings, the researchers suggest that reduced TCFL2 expression in adipocytes could lead to reduced glucose or lipid metabolism due to a high-fat diet (42). A study conducted on Chileans, the world’s largest consumers of sugar-sweetened beverages, suggested adverse effects in individuals who consume at least two sugar supplies per day. In addition, their findings link obesity, diabetes and genetic susceptibility involving the TCF7L2 and MTNR1B genes. The role of TCF7L2 in the development of these conditions may be because this gene influences the regulation of glucose metabolism through the WNT signaling pathway (43).

### Other genes related to obesity and metabolic pathways

The FABP2 gene codes for a protein that binds to FAs in the intestine and promotes active transport across the intestinal wall membrane; only the epithelial cells of the small intestine contain the intracellular protein. Hydrophobic FAs are transported from the plasma membrane to the ER *via* the aqueous cytosol by FABP2. In the ER, FAs are esterified with glycerol-3-phosphate (G3P) to form triglycerides. The triglycerides are packaged into chylomicrons, which circulate in the plasma. (131). A study confirmed that deletion of the FABP2 gene in mice results in weight gain and higher circulating triglyceride concentrations compared to wild-type mice (132).

*PPARG* and *CET* are two other genes involved in lipid metabolism. The PPARG gene encodes the nuclear receptor, which induces the proliferation of peroxisomes that regulate the transcription of several genes involved in the human body’s metabolism of lipids and carbohydrates, and inflammatory processes (133). The *CETP* gene is essential in lipid metabolism because it encodes the cholesterol ester transporter protein, which converts HDL cholesterol into LDL. High cholesterol levels and dietary fat intake cause an increase in CETP mRNA and protein concentration (134).

Finally, apolipoprotein A5 is the protein encoded by the *APOA5* gene. It plays a crucial role in regulating the level of triglycerides in the blood plasma (135).

## Future perspectives

The accelerated increase in the prevalence of obesity in Latin America makes it essential to develop nutrition-focused intervention strategies for the region focus on their eating patterns. Genetics and genomics in nutrition are tools that constitute the basis of understanding the genes pathways that are being affected due to nutrition leading to a greater susceptibility to obesity. In the future, it is intended to achieve personalized customization of the nutritional requirements of different populations and individuals based on the genetic inheritance of variants, ethnicity, and gene expression.

In this context, most of the studies in genetics, genomics, and epigenetics interactions with diet have been developed in European or North American countries. Therefore, association studies of genetic predisposition to obesity in the region are required. Moreover, the Latin American population is genetically different, marked by a mix of ethnic groups. Population in which the dietary improvements could potentially prevent deaths caused by obesity and the potential development of chronic diseases.

Furthermore, personalized medicine must be based on genetic evidence and environmental analysis as tools to prevent chronic diseases like obesity. Integrating new techniques or data obtained from genetics and genomics approaches in obesity could achieve a better quality of life and prompt response in the population of Latin America.

Lastly, nutrition programs should be promoted in Latin American environments where processed food and sugary drinks are a fundamental part of the diet. With the aim to make the population understand the relevance of healthy nutrition from an early age. Meanwhile, there are package labeling systems in several Latin American countries. However, it is still necessary to implement other measures that directly reach the consumer and create healthcare awareness through healthy eating.

In conclusion, several genes and their SNPs have been associated with obesity and obesity-related issues; however, only 7 of the 39 risk alleles have high frequencies in the mixed Latin American population. The risk alleles have been correlated with high total cholesterol, low HDL, vitamin D and B12 deficiency, and obesity predisposition. Furthermore, high-fat dietary behaviors could induce gene expression profiles related to insulin intolerance, lipolysis dysregulation, imbalanced energy expenditure, glucose dysregulation, and vitamin deficiency. Although the Latin American genetic background may not have an increased obesity genetic risk, the population should be aware of the dietary behavior in their environment to include all the necessary nutrients and avoid high-fat foods.

## Author contributions

PG-R, SC-U, and AZ: conceptualization and writing – review and editing. EP-C, RT-T, and VR-P: research. AZ and DS-R: supervision and conceptualization. All authors contributed to the article and approved the submitted version.
